# Supplementary material for: The causal relationship between the human gut microbiota and pyogenic arthritis: a Mendelian randomization study
Source: Front Cell Infect Microbiol. 2024 Nov 26;14:1452480. doi: 10.3389/fcimb.2024.1452480 (PMC11629706; doi:10.3389/fcimb.2024.1452480)
Supplement: Supplementary file 1 [file DataSheet1.zip › mendelian test/ebi-a-GCST90027595.csv_leave_one_out.pdf]

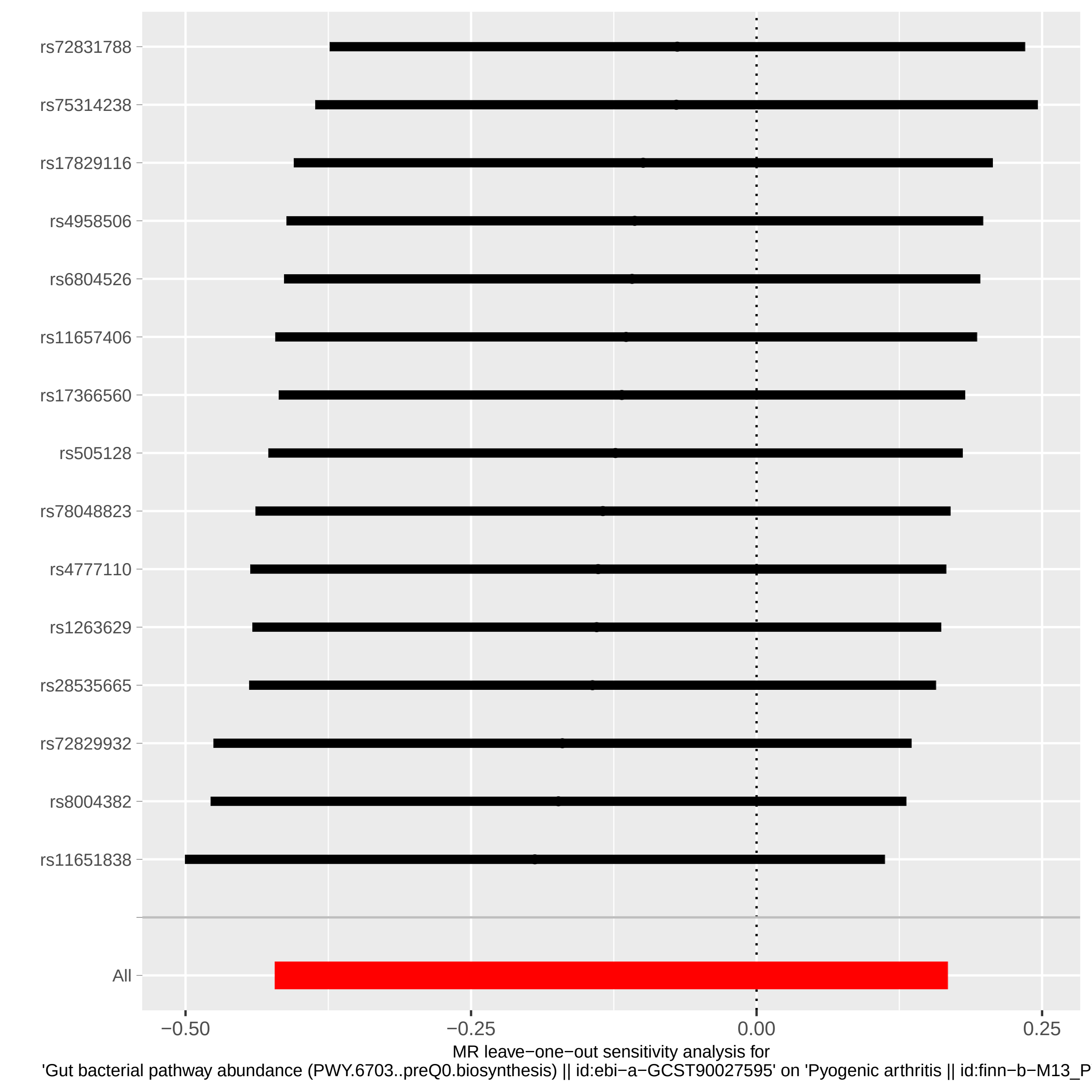
rs72831788
rs75314238
rs17829116
rs4958506
rs6804526
rs11657406
rs17366560
rs505128
rs78048823
rs4777110
rs1263629
rs28535665
rs72829932
rs8004382
rs11651838
All
−0.50
−0.25
0.00
0.25
MR leave−one−out sensitivity analysis for
'Gut bacterial pathway abundance (PWY.6703..preQ0.biosynthesis) || id:ebi−a−GCST90027595' on 'Pyogenic arthritis || id:finn−b−M13_P
